# Biomechanical Responses and Injury Characteristics of Knee Joints under Longitudinal Impacts of Different Velocities

**DOI:** 10.1155/2018/1407345

**Published:** 2018-08-05

**Authors:** Yan Xiong, Xueliang Zhao, Hongyi Xiang, Yunjiao Wang, Zhikang Liao, Xiyan Zhu, Hui Zhao

**Affiliations:** ^1^Department of Orthopedics, Daping Hospital and the Research Institute of Surgery, Third Military Medical University, Chongqing 400042, China; ^2^Institute for Traffic Medicine, State Key Laboratory of Trauma, Burns & Combined Wound, Third Military Medical University, Chongqing 400042, China

## Abstract

**Background and Objective:**

Knee joint collision injuries occur frequently in military and civilian scenarios, but there are few studies assessing longitudinal impacts on knee joints. In this study, the mechanical responses and damage characteristics of knee longitudinal collisions were investigated by finite element analysis and human knee impact tests.

**Materials and methods:**

Based on a biocollision test plateau, longitudinal impact experiments were performed on 4 human knee joints (2 in the left knee and 2 in the right knee) to measure the impact force and stress response of the bone. And then a finite element model of knee joint was established from the Chinese Visible Human (CVH), with which longitudinal impacts to the knee joint were simulated, in which the stress response was determined. The injury response of the knee joint-sustained longitudinal impacts was analyzed from both the experimental model and finite element analysis.

**Results:**

The impact experiments and finite element simulation found that low-speed impact mainly led to medial injuries and high-speed impact led to both medial and lateral injuries. In the knee joint impact experiment, the peak flexion angles were 13.8° ± 1.2, 30.2° ± 5.1, and 92.9° ± 5.45 and the angular velocities were 344.2 ± 30.8 rad/s, 1510.8 ± 252.5 rad/s, and 9290 ± 545 rad/s at impact velocities 2.5 km/h, 5 km/h, and 8 km/h, respectively. When the impact velocity was 8 km/h, 1 knee had a femoral condylar fracture and 3 knees had medial tibial plateau fractures or collapse fractures. The finite element simulation of knee joints found that medial cortical bone stress appeared earlier than the lateral peak and that the medial bone stress concentration was more obvious when the knee was longitudinally impacted.

**Conclusion:**

Both the experiment and FE model confirmed that the biomechanical characteristics of the injured femur and medial tibia are likely to be damaged in a longitudinal impact, which is of great significance for the prevention and treatment of longitudinal impact injuries of the knee joint.

## 1. Introduction

Knee joint injuries are commonly caused by traffic accidents, sports medicine, and falling from high altitudes [1–3]. As the main weight-bearing joint of the human lower extremities, the knee joint is characterized by various activities and complex anatomical and mechanical structures, and the mechanisms and biomechanical responses of the knee joint to injuries have been popular research topics [4–7]. A large number of experimental studies have been conducted worldwide studying injury mechanisms [8–10]. A recently published clinical study suggests that a single fracture-free blunt trauma will thicken the subchondral bone after injury [11], which is followed by chronic osteoarthrosis [12]. Currently, biomechanical studies concerning the knee joint are mainly carried out by performing impact tests in cadavers and studying knee fractures [13]. From impact tests with different bending angles of the knee joint, it was concluded that increases in the bending angle lead to increases in the fracture load. Furthermore, because of the knee's complicated anatomical structure, finite element models have been widely used in biomechanical studies of knee injuries [14]. However, in daily sports and military training [15], longitudinal impacts to the knee in a straight state often occur. In the above studies, the knee joint is mostly in a bent state [16], and studies assessing the mechanical response to longitudinal impacts to the knee in an extended state are relatively limited. As a result, the mechanical changes of the femoral-knee-tibiofibular structure and the mechanical responses of the accessory structures in the joint cavity while sustaining a longitudinal impact are still not clear. It is of great significance to explore the mechanisms and characteristics of longitudinal impact injuries with the knee in a straight position, as in sports and military injury scenarios. The purpose of the study, therefore, is to study the knee longitudinal impact injuries using both the impact experiment with cadaver knee samples and finite element model (FEM).

## 2. Materials and Methods

### 2.1. Knee Impact Experiment

This study was approved by the Ethics Committee of the Third Affiliated Hospital of the Third Military Medical University.

**Figure 1:**
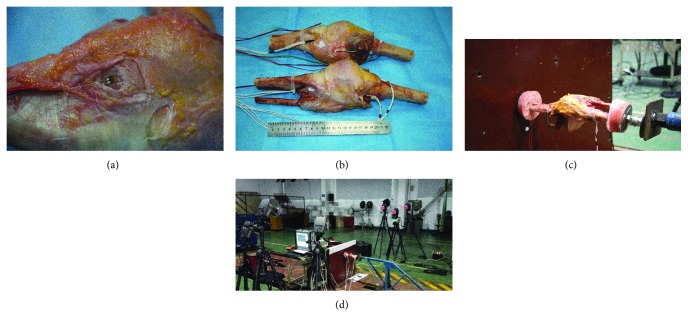
Photos of the experimental device to assess longitudinal impact on knee joints. (a) Method for implanting the knee joint strain sensing plate. (b) Condition after double knee electrode implantation. (c) Knee impact unit settings. (d) Traction track and Synergy version 5.0 data acquisition software.

Two unembalmed cadavers (4 knees) were used. Before the experiment, knee X-ray examinations were performed for the two specimens, and the specimen with bone injuries would be excluded. Knee joint specimens were obtained using a chainsaw, the joint capsule remained intact, and 15 cm of the proximal and distal knees was preserved. A screw was fixed to the femoral head with bone cement, and the screw was mounted on a rigid wall. The tibial end was fixed with a screw, and a force sensor (CL-YD-311A, Sinocera Piezotronics Inc., Jiangsu, China) was mounted to measure the impact force acting on the knee joint. Strain gauges (350 *Ω*) were attached to the medial and lateral condyles of the femur of the knee joint and the medial and lateral tibia, respectively, to measure the amount of strain in the bone (Figure 1). The specimens were put in a prone position during the knee impact experiment, that is, the patella was in a downward direction, the femur was connected with a fixed barrier, and the sled hit the tibia.

The impact experiments used a motor traction system, data acquisition system (Synergy C Rack, Hi-Techniques Inc., Madison, USA), and high-speed photography system (Phantom v12.1, Vision Research, Inc., Wayne, USA). The strain and impact force signals were sampled at a sampling frequency of 10 kHz. In the traction system, an impactor was mounted on a small sled, with a total mass of 65 kg. When the test was initiated, the sled was dragged and moved when it reached the specified speed. The moving impactor impacted the inferior portion of the knee. Each subject experienced the three impacts at the impact speed of 2.5 and 5.0 km/h and once at the impact speed of 8 km/h.

Following the impact, the responses of the knee joint were analyzed. In the study, *ε* is the strain value, determined by the formula $\varepsilon =\underset{L-0}{\lim}\left(△L/L\right)$. In this formula, *L* is the length before deformation, △*L* is the elongated length, the strain unit is 1 (the skeletal deformation is 0.1%), and strain *ε* is expressed as 10^−3^.

### 2.2. Finite Element Analysis

**Figure 2:**
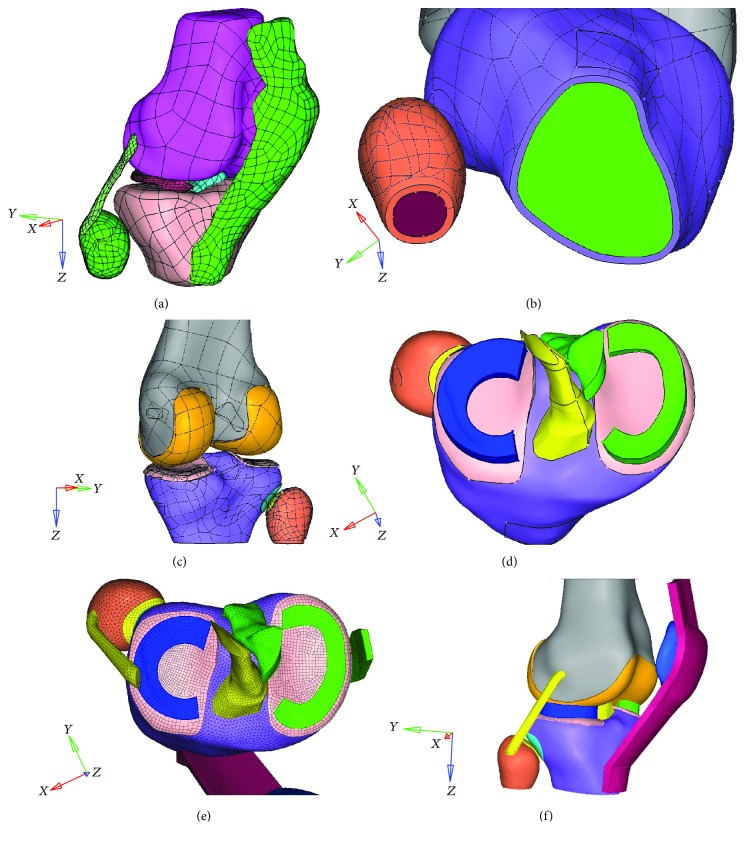
The development of the finite element model of the knee joint. (a) Initial inverse knee joint model showing a high-order surface after the smoothing processing. (b) The construction of cancellous bone and cortical bone boundaries; the inward bias of the cortical bone was set at 1.5 mm. (c) The articular cartilage thickness of the knee joint was set at 1 mm, and the cartilage thickness at the tibiofibular joint was set at 0.375 mm. (d) The reconstructed meniscus and cruciate ligament. (e) The mesh structure of the knee joint. (f) The completed finite element model of the knee joint.

Anatomical knee data from the Chinese Visible Human (CVH) of the Third Military Medical University was selected and imported into Amira® software to outline the boundaries of the knee tissues, exported in ASCII data format, and finally imported into HyperMesh® to establish the initial knee model (Figure 2); the data were processed using surface smoothing, and high-order surfaces were created that closely fit the smoothed elements. As the corpse specimen was maintained in a supine position for a long time, the relative positions of the cartilage ligaments in the joint had changed; hence, the distorted structures, such as the meniscus and ligaments, needed to be artificially constructed; the connections between the bone and the ligaments needed to be reconstructed; and a 15 cm long osteotomy needed to be applied.

Due to the different mechanical properties of cortical bone and cancellous bone, the boundary between the cortical bone and the cancellous bone needed to be accurately established. The thickness of the cortical bone was set at 1.5 mm. Then, on the outer surface of the cortical bone, the boundaries of each cartilage were outlined with reference to the anatomy. The outlined boundary was offset to the outside by an appropriate distance to generate the cartilage geometry of the body. The cartilage in the knee joint was set at a 1 mm thickness on the basis of references and anatomical structures, and the cartilage thickness in the tibiofibular joint was set at 0.375 mm. The medial meniscus is large and thin, with a “C” shape with a narrow front and a wide rear and an “O” shape on the outside [17]. The function of the meniscus is to stabilize the knee joint and transfer knee load [18]. The meniscus border was outlined in the tibial plateau cartilage. In addition, the connection between the cruciate ligament and the medial-lateral collateral ligament plays an extremely important role in the stability of the joint [19, 20]. The ligament model needed to be reconstructed based on the original ligament model and then connected with the bone [21].

During the mesh division, 1 mm grids were uniformly distributed on the surface of the meniscus. The grids were all quadrilateral and distributed in a concentric circular manner, and the surface grids were elongated into elements with a four-layer thickness. After dividing the meniscus mesh, the grids of the cartilage of the tibia that were connected with the meniscus were separated, and in this area, the nodes of the cartilage mesh were coincident with the nodes of the meniscus. Then, the grids on the medial collateral ligament, the patellar tendon, and the tibiofibular articular cartilage were divided. The cortical and cancellous bone regions were divided into 1 mm grids, which ensured more hexahedrons in the soft tissue, and the conjunction area between the soft tissue and bone shared the same nodes. Then, the grids on the femoral cartilage and lateral collateral ligament were divided. After finishing the grid division, the material properties, fixing, assembly, and loading constraints of the model were configured (Table 1), and the coefficient of friction was set as 0.1 [22]. Finally, a final finite element model of the knee joint was developed (Figure 2). Considering the brittle characteristics of the materials, the cortical bone was defined as failure when the stress reached 115 Mpa and the cancellous bone was considered failure when the stress reached 20 Mpa [23]. The model contained 490,978 units and 121,499 nodes.

## 3. Results

### 3.1. Knee Impact Experiment

The kinematic process were analyzed from the knee impact experiments, as shown in Table 2, in which at the speeds of 2.5, 5, and 8 km/h, the maximal rotational angles of the knee joint were 13.8 ± 1.2°, 30.2 ± 5.1°, and 92.9 ± 5.5°, respectively, while the angular velocities were 344.2 ± 30.8 rad/s, 1510.8 ± 252.5 rad/s, and 9290.0 ± 545.0 rad/s, respectively.  Table 3 showed that with increases in collision velocity, the bone strain amplitude peak values and average values increased significantly, with significant differences between each group (*p* < 0.05).

**Figure 3:**
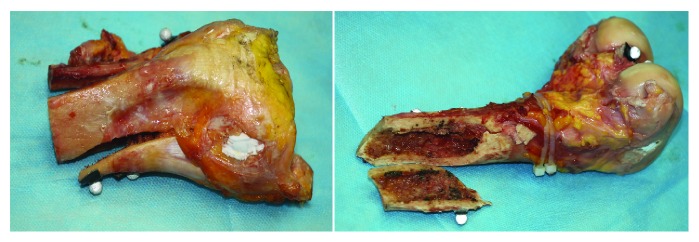
A fractured joint specimen at an impact speed of 8 km/h.

**Figure 4:**
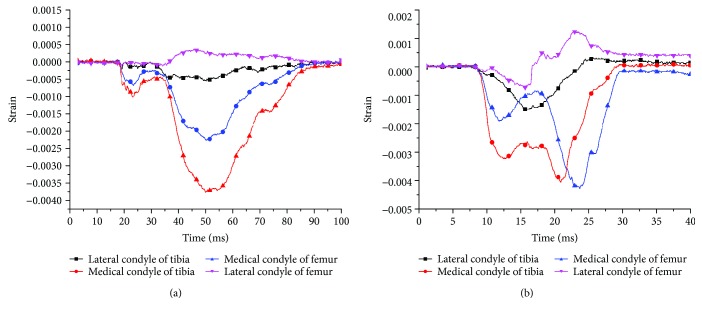
The strain curves of bones in the knee joints at the impact speeds of 2.5 (a) and 5 km/h (b).

No obvious damage was detected from the meniscus and ligaments at the impact speeds of 2.5 and 5.0 km/h, while at an impact velocity of 8 km/h, one knee of the two specimens showed fractures in the femoral shaft (Figure 3), and 3 knees showed fractures in the medial plateau, which were mostly split fractures and classified as Schatzker type IV. The collision strain curves at the impact rates of 2.5 km/h and 5 km/h were compared and showed that the velocity was positively related to the peak of the strain curve and the strain time (Figure 4).

### 3.2. Finite Element Simulation

**Figure 5:**
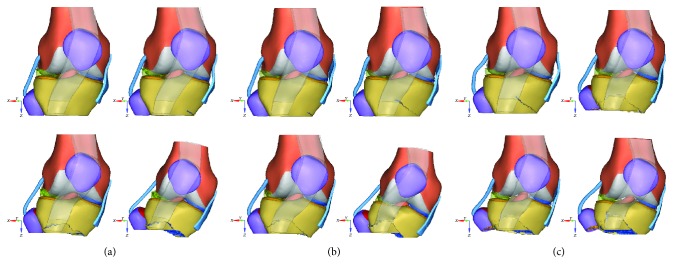
Knee kinematic process at the varied impact speeds ((a) 2.5 km/h; (b) 5.0 km/h; (c) 8.0 km/h).

Figure 5 showed the kinematic process and stress distribution with the failure mode, in which at an impact velocity of 2.5 km/h, for example, the bottom of the medial tibia first showed failure and then the failure extended to the center of the tibia until the entire tibial plateau was fractured. The knee was further inverted throughout the impact process. Before the fracture, the maximal varus angle of the femur was 12.28°. At an impact velocity of 5 km/h, the bottom of the medial tibia first showed cracks, and then, the crack extended to the center of the tibia until the entire tibial plateau was fractured. Compared to the 2.5 km/h impact velocity, the destruction was more intense at an impact velocity of 5 km/h. The figure also showed cracks at the bottom of the medial tibia at an impact speed of 8 km/h, followed by more intense fractures, making the entire fixed plane to show a comminuted fracture.

**Figure 6:**
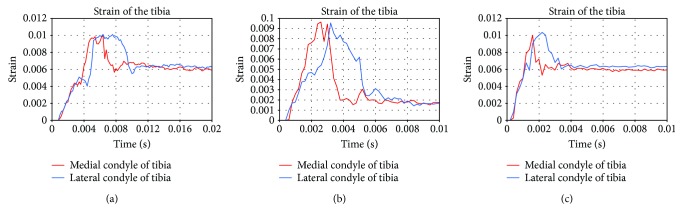
The strain response of medial and lateral tibial strain curves at the varied impact speeds ((a) 2.5 km/h; (b) 5.0 km/h; (c) 8.0 km/h).

**Figure 7:**
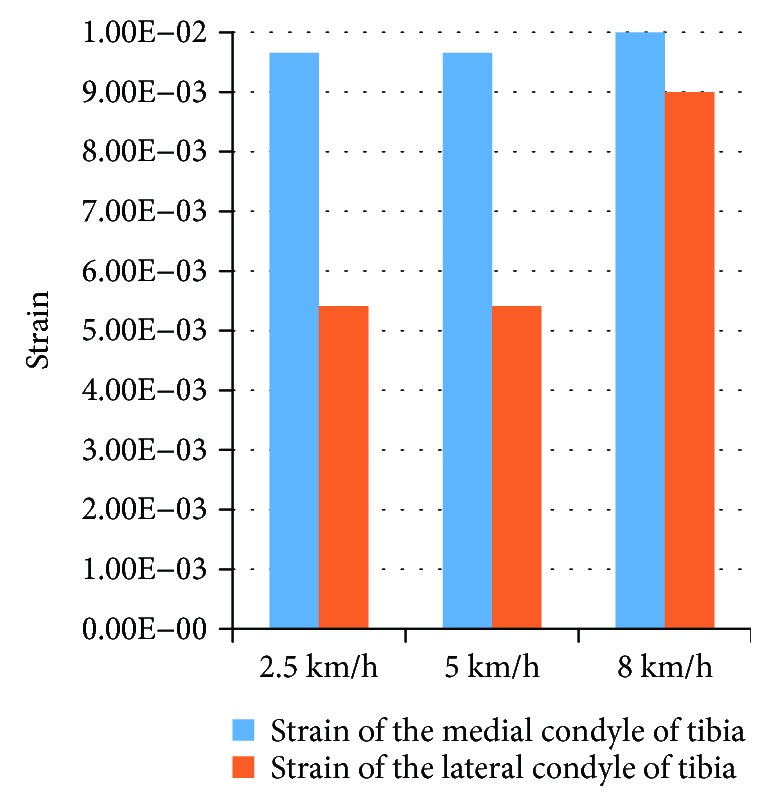
Strain peaks of the medial tibia at the varied impact speeds.

Figure 6 shows the strain of the bone impacted at the varied speeds. The curves indicated that the strain peak occurred first in the medial tibia and that the bigger the impact velocity, the earlier the peak appeared. The data in Figure 7 indicated that the medial strain of the tibia was still at a relatively small level when the peak of the strain reached at the medial tibia. Due to the destruction of the medial tibia, the maximum strain was the strain value at breakage, and the strain was essentially the same.

**Figure 8:**
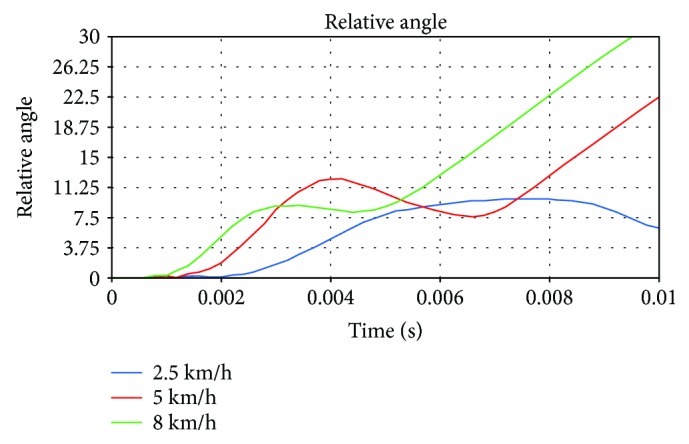
Curves of the varus angle at different impact speeds.

**Figure 9:**
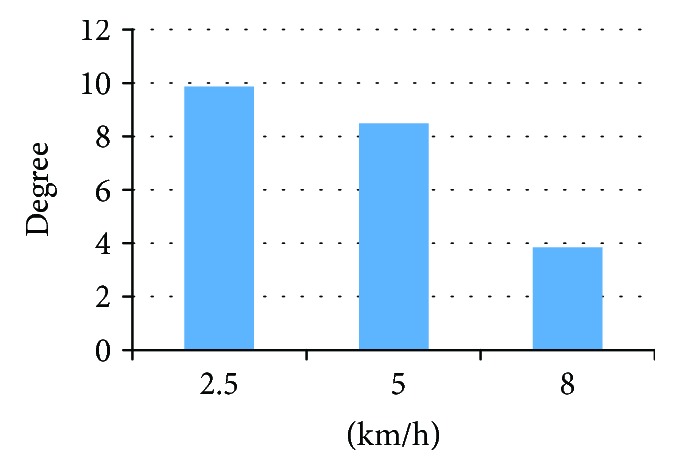
The maximum tilting angle at different velocities before fractures occurred.

**Figure 10:**
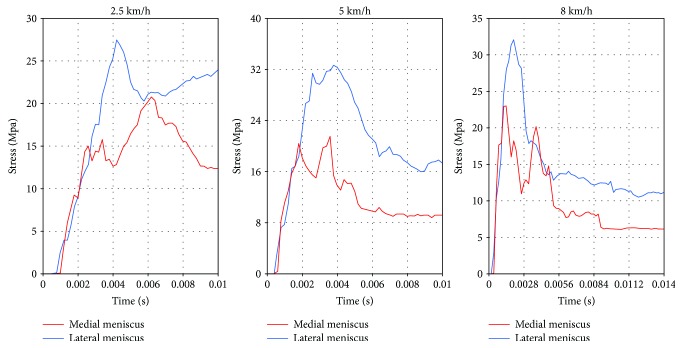
Meniscus stress curves at the varied impact speeds.

The simulation for the longitudinal impacts to knee joint exhibited varied degrees of varus at various impact speeds as shown in Figure 8. The maximum varus angles under the three impact velocities before the fracture are shown in Figure 9. As shown in Figure 10, before the initial crack, the stress on both meniscuses increased rapidly, and the medial side was slightly larger than the lateral side. When the crack occurred in the medial tibial base, the stress on the medial meniscus increased slowly. At this time, the lateral meniscus stress was greater than that of the inside. The meniscus stress had the same tendencies at all the speeds.

## 4. Discussion

Impacting injury to the knee joint is most commonly seen in traffic injuries [27] or sports injuries [28]. Most studies have been conducted under knee bending conditions [29], and there are relatively few studies on the mechanisms and characteristics of impact injury, especially for knee injuries induced by longitudinal impacts. In this study, knee specimens obtained from unembalmed cadavers were used, and the impact tests were conducted in a straight knee position to simulate the occurrence of human falling injuries or military training injuries and to explore the biomechanical characteristics of longitudinal impacts on the knee joint [30].

Previous experimental studies of impact injuries have mostly focused on the knee injury mechanisms in traffic accidents [27]. Bose et al. [31] conducted collision tests on 40 knee specimens to simulate knee impact injuries in traffic accidents to explore the threshold of the knee valgus angle and shear displacement. Ruan et al. [4] showed that in frontal collisions between motor vehicles and pedestrians, the knee flexion angle, impact direction, and shape of the contact surface were all factors that affected the severity of the injury.

Our knee longitudinal impact experiment found that the speed of the impact unit was positively related to the knee flexion speed and the angle of flexion, indicating that the human knee buffers longitudinal impacts on the knee through knee flexion when falling from a high level, which may be related to increased contact area and longer force duration resulting from meniscus deformation during knee flexion [32]. At the same time, the initial flexion angular velocity is smaller during the process of knee flexion, and the angular velocity increases rapidly after a 30° flexion, which is related to the stress characteristics of the knee flexion process. We assume that the bone of the femoral and tibial medial and lateral condyle is the same, and knee stress findings showed that under the same impact velocity, the medial tibial plateau and the medial femoral condyle had greater deformation, stress range, and peak values. Our study found that in impact experiments using the cadaveric specimen, the knee injury but fracture was difficult to detect while Pedersen et al. [33] considered that among the longitudinal knee impact injuries, bone contusion sizes combined with time of persistence are likely better measures of joint injury severity than isolated bone contusion volume.

In addition, studies [34] have shown that the bone mineral density of the medial tibial plateau is lower than that of the lateral side, which is one of the reasons that the medial side is more vulnerable. Yukata et al. [35] studied stress fractures of the tibial plateau and found that all stress fractures occurred in the medial plateau and that the fracture location was related to the posterior tilt of the medial tibial plateau. In our study, we found that in the knee longitudinal collision injury test, a longitudinal low-speed impact often led to an inner knee injury and a high-speed collision often resulted in both medial and lateral knee injuries. In actual scenarios, there were several injury risks associated with the longitudinal knee impact injuries, that is, male patients, age < 30 years, and particularly patients who sustained a contact injury, and as a result, special attention is therefore necessary in those patients and early referral to magnetic resonance imaging and/or arthroscopy is recommended to allow meniscus repair in a timely manner [36].

In recent years, finite element analysis has been widely used, applied in the establishment of joint models, and used to simulate joint stress changes under different conditions, such as changes in the stress and strain of the femur and tibia after hip and knee replacements [3, 37–41]. Compared to experiments, the simulations conducted using finite element models have advantages such as high efficiency and noninvasiveness, and they allow for the expedient study of the mechanical responses of the knee joint. Using CVH-based knee anatomy data to build the finite element model could mimic better accurate knee anatomic structure, while it may reconstruct the meniscus and related ligaments in the knee and ensures the stability of the knee in the sagittal plane [42]. In the simulated impact experiment, the simulated impact unit used an average 65 kg body weight at the varied impact speeds to maximally simulate the characteristics of stress and strain in the knee joint during the longitudinal impact process. The results from the simulation and experiment showed that the knee begins to rotate when a longitudinal impact is loaded, and with an increase in the impact energy, the angle of the rotation increased. Our results found that in addition to rotation, the bone sustained a strain when knee was contacted. Huang et al. also showed that a knee joint finite element model could effectively simulate the characteristics of knee injury caused by contact in a car accident [43].

**Figure 11:**
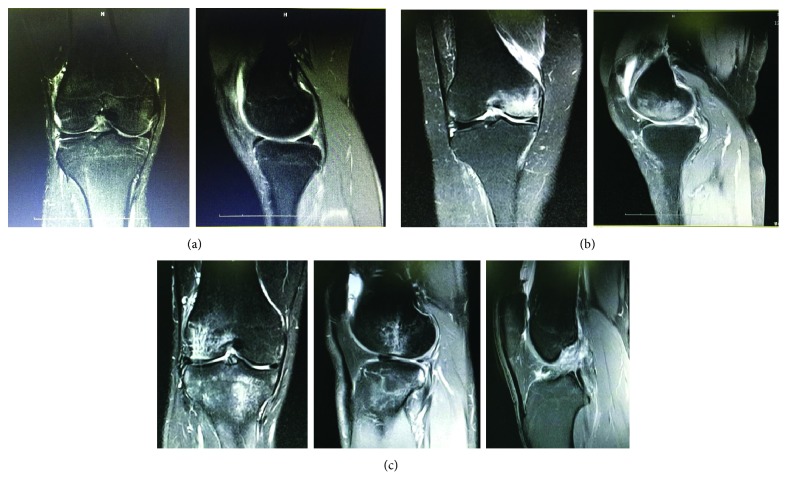
(a) Case 1: male, 21 years old, long distance runner, right knee pain, and swelling after knee longitudinal impact injury in one long-distance race. MRI showed that medial femur and medial tibia had mild bone bruise, bone marrow edema, and mild joint effusion. (b) Case 2: male, 77 years old, right knee pain, and swelling after one falling longitudinal impact injury at one meter high. MRI showed widely that medial femoral condyle had bone marrow injury and edema. Medial tibia had mild bone bruise and joint effusion. (c) Case 3: male, 22 years old, soldier, right knee pain, swelling, and movement dysfunction after knee longitudinal impact injury in one military training. MRI showed widely that medial tibia had bone marrow edema, meniscus injury, and rupture of anterior cruciate ligament.

Makinejad et al. [44] studied the mechanisms of longitudinal impacts on the knee joint, which was similar to our study. They investigated the stress and deformation processes of the knee joint during the falling process from different heights and concluded that longitudinal impact to the knee joint is more likely to cause damage, but the distribution of the injury sites is not yet clear. Dong et al. [45] kept the knee straight and compressed 1150 N on the knee joint and concluded that a meniscus tear and partial meniscectomy can accelerate the knee joint injury, which had a significant effect on the pressure peaks and shearing force of medial meniscus and cartilage, which is consistent with the results of this study. Our study found that there was a greater risk of injury to the medial meniscus, tibia, and medial femoral condyle of the knee when the knee collision occurred while the knee was straight; that longitudinal low-speed impact mainly led to inner knee injury; and that high-speed impact led to both lateral and medial knee injuries. Some studies [46] have shown that incorrect running posture can cause varus deformities of the knee and even cause “O”-shaped legs. The stress distribution and damage characteristics of longitudinal impact injuries to the knee joint in the corpse specimens were consistent with those of the three-dimensional finite element analysis and coincided with the medial stress fractures seen in knee joints in clinical practice, as shown in Figure 11. To prevent knee arthritis [47], one should also consider the more severe medial stress damage caused by repeated longitudinal impact injuries to the knee joint during daily life; thus, the incidence of knee osteoarthritis with genu varum is higher. Moreover, in clinical practice, in the early stages of knee osteoarthritis, using a lateral fibular osteotomy to reduce the medial knee stress can achieve good clinical efficacy [48].

## 5. Limitations

In this study, the collision experiment used knee specimens for in vitro experiments, which have both advantages [13] and shortcomings. As the body specimens enrolled in the study were old, the structure and strength may have been different from younger specimens. Consequently, the responses derived from the study may represent those occurring in seniors. The number of specimens used in this study was small, and the specimens experienced more than one impact. In the impact experiment, however, the cumulative effects of impact at different speeds were not considered. The establishment of the knee finite element model was based on CVH data rather than the specimens, while the difference between the tested specimens and the finite element model was not considered, and the further validation has not been done in the present study.

## 6. Conclusion

The three-dimensional finite element analysis and impact experiments showed that the stress response characteristics of the femur and the medial condyle of the tibia are more prone to damage under different longitudinal impact velocities. Longitudinal low-speed collisions often lead to inner knee injury, and high-speed collisions often result in both medial and lateral knee injuries. This study can play an important role in providing key data for the prevention and treatment of longitudinal collision injuries of the knee joint.

## Figures and Tables

**Table 1:** Material assignment of the finite element model of the knee joint.

Materials	Model	Parameters	Reference
Cortical bone	ISO ILASTIC	*E* = 12,000, *u* = 0.3	[24]
Cancellous bone	ISO ILASTIC	*E* = 400, *u* = 0.3	[24]
Articular cartilage	ISO ILASTIC	*E* = 100, *u* = 0.3	[25]
Meniscus	ISO ILASTIC	Incompressible, C10 = 2.67, C01 = 0.667 (*E* = 20, *u* = 0.49)	[25]
Ligament	ISO ILASTIC	*E* = 10, *u* = 0.3	[26]

**Table 2:** Angle changes in knees at different velocities.

Impact speed	Range of motion				Mean value
	N1 left	N1 right	N2 left	N2 right	
	MAX° w(rad/s)	MAX° w(rad/s)	MAX° w(rad/s)	MAX° w(rad/s)	MAX° w(rad/s)
2.5 km/h	15.7 392.5	13.6 340.0	14.1 352.5	12.2 305.0	13.9 ± 1.0 347.5 ± 25.0
5.0 km/h	26.2 1325.0	20.6 1030.0	35.2 1760	32.4 1620	28.6 ± 5.2 1433.75 ± 256.25
8 km/h	94.1 9410.0	98.6 9860.0	83.2 8320.0	88.6 8860.0	91.1 ± 5.2 9112.5 ± 522.5

MAX°: maximum deformation angle of the knee joint; W: A/T; A: angle change; T: time to maximum deformation.

**Table 3:** Strain and impact force of knee joint.

Specimen	Impact speed	Strain				Impact force (N)
		Medial condyle of femur	Lateral condyle of femur	Medial condyle of tibia	Lateral condyle of tibia	
		Peak value (×10^−6^)	Peak value (×10^−6^)	Peak value (×10^−6^)	Peak value (×10^−6^)	Peak value
N1	2.5 km/h	2116.3 ± 31.3	1835.5 ± 32.1	3258.3 ± 53.6	1357.5 ± 24.3	2164.4 ± 421.2
	5.0 km/h	4815.7 ± 83.7	3865.4 ± 63.2	7103.1 ± 97.3	1394.6 ± 23.1	3729.5 ± 127.3
	8 km/h	6581.4 ± 149.6	5321.5 ± 161.2	12634.6 ± 235.6	7653.2 ± 81.6	5639.2 ± 653.8

N2	2.5 km/h	1534.6 ± 30.1	1235.2 ± 16.3	3125.4 ± 14.3	2525.1 ± 21.3	2310.2 ± 324.5
	5.0 km/h	4635.2 ± 21.3	3765.4 ± 59.3	7058.5 ± 85.2	2312.6 ± 25.1	4720.3 ± 797.8
	8 km/h	6638.2 ± 152.6	4521.5 ± 122.6	11562.3 ± 211.5	8426.9 ± 74.2	6591.2 ± 336.2

N3	2.5 km/h	2015.5 ± 21.6	1845.3 ± 30.5	3325.1 ± 56.4	1325.3 ± 21.1	2246.1 ± 399.2
	5.0 km/h	4526.1 ± 78.6	3567.4 ± 61.2	6829.5 ± 88.5	3356.2 ± 21.0	5528.7 ± 639.4
	8 km/h	7229.6 ± 126.5	5638.2 ± 121.6	11561.2 ± 253.6	8465.3 ± 75.3	8639.2 ± 556.2

N4	2.5 km/h	1985.6 ± 22.9	1562.8 ± 16.2	3122.4 ± 33.2	1242.6 ± 22.6	2256.8 ± 413.5
	5.0 km/h	4562.3 ± 56.9	3356.2 ± 22.9	5687.2 ± 88.6	1234.1 ± 56.3	4562.5 ± 648.3
	8 km/h	6675.3 ± 133.5	5013.2 ± 155.2	8965.8 ± 225.1	6628.1 ± 85.3	5864.2 ± 655.2

## Data Availability

The data used to support the findings of this study are available from the corresponding author upon request.

## References

[1] Nie B., Crandall J. R., Panzer M. B. (2016). Computational investigation of the effects of knee airbag design on the interaction with occupant lower extremity in frontal and oblique impacts. *Traffic Injury Prevention*.

[2] Cameron K. L., Peck K. Y., Thompson B. S., Svoboda S. J., Owens B. D., Marshall S. W. (2015). Reference values for the Marx activity rating scale in a young athletic population: history of knee ligament injury is associated with higher scores. *Sports Health: A Multidisciplinary Approach*.

[3] Filipovic N., Vulovic R., Peulic A., Radakovic R., Kosanic D., Ristic B. (2009). Noninvasive determination of knee cartilage deformation during jumping. *Journal of Sports Science & Medicine*.

[4] Ruan J. S., el-Jawahri R., Barbat S., Rouhana S. W., Prasad P. (2008). Impact response and biomechanical analysis of the knee-thigh-hip complex in frontal impacts with a full human body finite element model. *Stapp Car Crash Journal*.

[5] Multanen J., Rantalainen T., Kautiainen H. (2017). Effect of progressive high-impact exercise on femoral neck structural strength in postmenopausal women with mild knee osteoarthritis: a 12-month RCT. *Osteoporosis International*.

[6] Mohd Sharif N. A., Goh S. L., Usman J., Wan Safwani W. K. Z. (2017). Biomechanical and functional efficacy of knee sleeves: a literature review. *Physical Therapy in Sport*.

[7] Thangavel P., Vidhya S., Li J., Chew E., Bezerianos A., Yu H. Biomechanical effects of robot assisted walking on knee joint kinematics and muscle activation pattern.

[8] Carter J. C., Sturnick D. R., Vacek P. M. (2017). Relationship between geometry of the extensor mechanism of the knee and risk of anterior cruciate ligament injury. *Journal of Orthopaedic Research*.

[9] Cetinkaya E., Aydin C. G., Akman Y. E. (2015). A rare knee extensor mechanism injury: vastus intermedius tendon rupture. *International Journal of Surgery Case Reports*.

[10] Kiapour A. M., Quatman C. E., Goel V. K., Wordeman S. C., Hewett T. E., Demetropoulos C. K. (2014). Timing sequence of multi-planar knee kinematics revealed by physiologic cadaveric simulation of landing: implications for ACL injury mechanism. *Clinical Biomechanics (Bristol, Avon)*.

[11] Fischenich K. M., Button K. D., Coatney G. A. (2015). Chronic changes in the articular cartilage and meniscus following traumatic impact to the lapine knee. *Journal of Biomechanics*.

[12] Ewers B. J., Jayaraman V. M., Banglmaier R. F., Haut R. C. (2000). The effect of loading rate on the degree of acute injury and chronic conditions in the knee after blunt impact. *Stapp Car Crash Journal*.

[13] Maletsky L., Shalhoub S., Fitzwater F. (2016). In vitro experimental testing of the human knee: a concise review. *The Journal of Knee Surgery*.

[14] Kiapour A., Kiapour A. M., Kaul V. (2014). Finite element model of the knee for investigation of injury mechanisms: development and validation. *Journal of Biomechanical Engineering*.

[15] Divine J. (2012). Exercise training to prevent anterior knee pain in military recruits. *Clinical Journal of Sport Medicine*.

[16] Torres R., Ferreira J., Silva D., Rodrigues E., Bessa I. M., Ribeiro F. (2017). Impact of patellar tendinopathy on knee proprioception: a cross-sectional study. *Clinical Journal of Sport Medicine*.

[17] Kohn D., Moreno B. (1995). Meniscus insertion anatomy as a basis for meniscus replacement: a morphological cadaveric study. *Arthroscopy*.

[18] Fox A. J. S., Wanivenhaus F., Burge A. J., Warren R. F., Rodeo S. A. (2015). The human meniscus: a review of anatomy, function, injury, and advances in treatment. *Clinical Anatomy*.

[19] Varelas A. N., Erickson B. J., Cvetanovich G. L., Bach B. R. (2017). Medial collateral ligament reconstruction in patients with medial knee instability: a systematic review. *Orthopaedic Journal of Sports Medicine*.

[20] Domnick C., Frosch K. H., Raschke M. J. (2017). Kinematics of different components of the posterolateral corner of the knee in the lateral collateral ligament-intact state: a human cadaveric study. *Arthroscopy: The Journal of Arthroscopic & Related Surgery*.

[21] Djoudi F. (2013). 3D reconstruction of bony elements of the knee joint and finite element analysis of total knee prosthesis obtained from the reconstructed model. *Journal of Orthopaedics*.

[22] Arun M. W. J., Umale S., Humm J. R., Yoganandan N., Hadagali P., Pintar F. A. (2016). Evaluation of kinematics and injuries to restrained occupants in far-side crashes using full-scale vehicle and human body models. *Traffic Injury Prevention*.

[23] Element L. L. C. (2014). User manual: M50 occupant version 4.3 for Ls-Dyna. *Global Human Body Model Consortium-owned GHBMC Model*.

[24] Zhang Q. H., Teo E. C., Ng H. W., Lee V. S. (2006). Finite element analysis of moment-rotation relationships for human cervical spine. *Journal of Biomechanics*.

[25] Pitzen T. R., Matthis D., Barbier D. D., Steudel W. I. (2002). Initial stability of cervical spine fixation: predictive value of a finite element model. Technical note. *Journal of Neurosurgery*.

[26] Schmidt H., Heuer F., Simon U. (2006). Application of a new calibration method for a three-dimensional finite element model of a human lumbar annulus fibrosus. *Clinical Biomechanics (Bristol, Avon)*.

[27] Nagasaka K., Mizuno K., Tanaka E. (2003). Finite element analysis of knee injury risks in car-to-pedestrian impacts. *Traffic Injury Prevention*.

[28] Yoganandan N., Banerjee A., Hsu F. C. (2016). Deriving injury risk curves using survival analysis from biomechanical experiments. *Journal of Biomechanics*.

[29] Atkinson P. J., Haut R. C. (2001). Injuries produced by blunt trauma to the human patellofemoral joint vary with flexion angle of the knee. *Journal of Orthopaedic Research*.

[30] Atkinson P. J., Haut R. C. (2001). Impact responses of the flexed human knee using a deformable impact interface. *Journal of Biomechanical Engineering*.

[31] Bose D., Bhalla K. S., Untaroiu C. D., Ivarsson B. J., Crandall J. R., Hurwitz S. (2008). Injury tolerance and moment response of the knee joint to combined valgus bending and shear loading. *Journal of Biomechanical Engineering*.

[32] Besier T. F., Draper C. E., Gold G. E., Beaupré G. S., Delp S. L. (2005). Patellofemoral joint contact area increases with knee flexion and weight-bearing. *Journal of Orthopaedic Research*.

[33] Pedersen D. R., el-Khoury G. Y., Thedens D. R., Saad-Eldine M., Phisitkul P., Amendola A. (2017). Bone contusion progression from traumatic knee injury: association of rate of contusion resolution with injury severity. *Open Access Journal of Sports Medicine*.

[34] Lo G. H., Zhang Y., McLennan C. (2006). The ratio of medial to lateral tibial plateau bone mineral density and compartment-specific tibiofemoral osteoarthritis. *Osteoarthritis and Cartilage*.

[35] Yukata K., Yamanaka I., Ueda Y. (2017). Medial tibial plateau morphology and stress fracture location: a magnetic resonance imaging study. *World Journal of Orthopedics*.

[36] Feucht M. J., Bigdon S., Bode G. (2015). Associated tears of the lateral meniscus in anterior cruciate ligament injuries: risk factors for different tear patterns. *Journal of Orthopaedic Surgery and Research*.

[37] Pfeiffer F. M. (2016). The use of finite element analysis to enhance research and clinical practice in orthopedics. *The Journal of Knee Surgery*.

[38] Chang C. Y., Rupp J. D., Reed M. P., Hughes R. E., Schneider L. W. (2009). Predicting the effects of muscle activation on knee, thigh, and hip injuries in frontal crashes using a finite-element model with muscle forces from subject testing and musculoskeletal modeling. *Stapp Car Crash Journal*.

[39] Beidokhti H. N., Janssen D., van de Groes S., Verdonschot N. (2018). The peripheral soft tissues should not be ignored in the finite element models of the human knee joint. *Medical & Biological Engineering & Computing*.

[40] Huang S. C. (2017). The study of stresses characteristic of contact mechanism in total knee replacement using two-dimensional finite element analysis. *Bio-medical Materials and Engineering*.

[41] Sun Z. H., Liu Y. J., Li H. (2014). Femoral stress and strain changes post-hip, -knee and -ipsilateral hip/knee arthroplasties: a finite element analysis. *Orthopaedic Surgery*.

[42] Ren D., Liu Y., Zhang X., Song Z., Lu J., Wang P. (2017). The evaluation of the role of medial collateral ligament maintaining knee stability by a finite element analysis. *Journal of Orthopaedic Surgery and Research*.

[43] Huang W. H., Huang P., Li Z. D. (2014). 3D finite element model of human knee injuries in the traffic accident. *Fa Yi Xue Za Zhi*.

[44] Makinejad M. D., Osman N. A., Abas W. A., Bayat M. (2013). Preliminary analysis of knee stress in full extension landing. *Clinics (São Paulo, Brazil)*.

[45] Dong Y., Hu G., Dong Y., Hu Y., Xu Q. (2014). The effect of meniscal tears and resultant partial meniscectomies on the knee contact stresses: a finite element analysis. *Computer Methods in Biomechanics and Biomedical Engineering*.

[46] Asadi K., Mirbolook A., Heidarzadeh A., Mardani Kivi M., Emami Meybodi M. K., Rouhi Rad M. (2015). Association of soccer and genu varum in adolescents. *Trauma Monthly*.

[47] Lopez-Olivo M. A., Ingleshwar A., Volk R. J. (2018). Development and pilot testing of multimedia patient education tools for patients with knee osteoarthritis, osteoporosis, and rheumatoid arthritis. *Arthritis Care & Research*.

[48] Yang Z. Y., Chen W., Li C. X. (2015). Medial compartment decompression by fibular osteotomy to treat medial compartment knee osteoarthritis: a pilot study. *Orthopedics*.

